# Serologic Evidence of Human Exposure to Bat-Borne Zoonotic Paramyxoviruses, Cambodia

**DOI:** 10.3390/v17081146

**Published:** 2025-08-21

**Authors:** Neil Mittal, Spencer L. Sterling, Phireak Hip, Dolyce H. W. Low, Piseth Ly, Menghou Mao, Pidor Ouch, Adrian C. Paskey, Lianying Yan, Alan Hitch, Gavin J. D. Smith, Jeffery Hertz, Andrew G. Letizia, Ian H. Mendenhall, Eric D. Laing

**Affiliations:** 1 Department of Microbiology and Immunology, School of Medicine, Uniformed Services University, Bethesda, MD 20814, USA; 2 Henry M. Jackson Foundation for the Advancement of Military Medicine, Bethesda, MD 20817, USA; 3 United States Naval Medical Research Unit INDO PACIFIC, Singapore 109679, Singapore; 4 Programme in Emerging Infectious Diseases, Duke-National University of Singapore Medical School, Singapore 169857, Singapore; 5 Naval Medical Research Command, Silver Spring, MD 20910, USA; 6 Museum of Wildlife and Fish Biology, Department of Wildlife, Fish and Conservation Biology, University of California, Davis, CA 95616, USA; 7 Department of Data Science, Harrisburg University of Science and Technology, Harrisburg, PA 17101, USA

**Keywords:** henipaviruses, Cambodia, Cedar virus, Menangle virus, surveillance, multiplex serology

## Abstract

Fruit bats in the genus *Pteropus* are the natural reservoirs for zoonotic paramyxoviruses, notably henipaviruses and pararubulaviruses, which are found across Southeast Asia and Oceania. The genetic and antigenic diversity of viruses in both genera, and region specificity, are ill-defined, limiting health security measures aimed at minimizing spillover. For example, Nipah virus has been isolated from bats in the Battambang province of western Cambodia, and surveys suggest bat foraging behaviors occur in close proximity to human settlements. However, there have been no historical cases of Nipah virus in Cambodia. Here, we use a multiplex microsphere immunoassay to identify cryptic human exposure to selected henipaviruses and pararubulaviruses in Cambodia. Convalescent human sera from persons presenting with acute respiratory illness were screened to detect the presence or absence of antibodies reactive with attachment glycoprotein antigens from Nipah virus, Hendra virus, Cedar virus, and Ghana virus, and a hemagglutinin-neuraminidase antigen from Menangle virus. In this sero-survey, we detected antibodies that were specifically reactive with Cedar virus and Menangle virus, including one serum sample that neutralized a recombinant Cedar virus. Additionally, we detected a pattern of cross-reactivity with Hendra virus, Cedar virus, and Ghana virus, suggesting previous infection by an antigenically-related henipavirus. We did not detect high antibody reactivity with the NiV glycoprotein. Future studies should expand serological surveillance for these transboundary pathogens, including genetic surveillance to aid in henipavirus discovery, and focused biosurveillance where interfaces with livestock and humans occur.

## 1. Introduction

Over the last 100 years, the majority of viral pandemics have been caused by zoonotic viruses, those with a transmission route that can be traced back to a wildlife host. Biosurveillance within a One Health framework is frequently utilized as a health security tool to detect biohazards such as zoonotic viruses, and to generate epidemiological surveillance data aimed at minimizing the biothreat of spillover by identifying spatial and temporal risks associated with human exposure. Directly identifying virus spillover from wildlife reservoirs to human populations has proven challenging. Thus, targeted surveillance of humans at animal–human interfaces where there are perceived risks for exposure to zoonotic viruses, for example, occupational risk associated with raising and butchering of livestock, e.g., Nipah virus in Malaysia and Singapore [1], and Reston virus in the Philippines [2], may improve early detection of spillover events. Additionally, strengthening diagnostic capacity and investing in surveillance of human populations presenting to clinics with disease will likely increase the chances of detecting early evidence of spillover because health-care seeking behavior should be unaffected by whether the virus is a common causative agent of respiratory illness or a rare zoonotic virus.

Of the emerging zoonotic viruses, several are highly virulent in both humans and livestock populations. Nipah virus (NiV) is a bat-borne zoonotic RNA virus responsible for outbreaks with case fatality rates of respiratory illness and encephalitis reaching 70–100% in some outbreaks. NiV was discovered following disease outbreaks among pigs and pig-farm workers in Malaysia and Singapore, 1999 [3,4]. Subsequently, a Bangladesh strain of NiV was identified after an outbreak of fatal encephalitis in Bangladesh in 2004 [5] and was the cause of undiagnosed febrile illness and encephalitis in Bangladesh from 2001 to 2003 and in 2001 in West Bengal, India [6]. Annual outbreaks in Bangladesh are linked to the consumption of NiV-contaminated unpasteurized date palm [7,8,9]. Expansion of afflicted areas outside of Bangladesh was noted following a zoonotic event of NiV-like disease in 2015 in the Philippines [10], and near yearly NiV outbreaks in Kerala, southern India, starting in 2018, further highlighted gaps in our understanding of the at-risk geographies and populations for NiV emergence [11,12,13,14,15]. NiV has been detected in resident populations of fruit bats in both Thailand and Cambodia [16,17,18,19], although to date there have been no recorded outbreaks.

Beyond NiV, diverse henipaviruses and related paramyxoviruses in the genus *Pararubulavirus* have been detected in flying foxes and fruit bats found in Africa, Asia, and Oceania. Henipaviruses, Hendra virus (HeV), and Cedar virus (CedV) are hosted by *Pteropus* species of fruit bats native to Australia [20,21]. Like NiV, HeV is a priority pathogen and the causative agent of fatal respiratory illness and meningitis in livestock and humans [22]. On the other hand, CedV lacks key virulence factors, is not pathogenic in animal models [21], and lacks empirical evidence of zoonosis. The first African henipavirus, Ghana virus (GhV), was discovered in a species of fruit bats (*Eidolon helvum*) and sequences of other novel African henipaviruses have been identified in related *Eidolon* species and Egyptian fruit bats [23,24,25]. Pararubulaviruses, which are found in African and Asiatic fruit bats are characterized by a surface attachment glycoprotein with both hemagglutinin and neuraminidase activity, and often co-circulate in populations of fruit bats that host henipaviruses [26,27,28]. In Australia, Menangle virus (MenV) is a zoonotic paramyxovirus linked with flying foxes that causes outbreaks of congenital abnormalities and stillborn deaths in pigs, and influenza-like illness with rash presentations in humans [29,30,31,32,33]. A closely related pararubulavirus, Tioman virus, was identified in samples collected from flying foxes near Peninsular Malaysia, and causes a non-severe illness in pigs [34,35].

The geographic distribution of these viruses and their relatives is largely uncharacterized. Known and novel viruses are most often detected once outbreaks reach a critical size. To understand the biothreat risk posed by NiV and other zoonotic paramyxoviruses, we retrospectively tested archived human serum samples collected in an acute febrile illness study in Cambodia. Viremia is short-lived compared to memory responses induced during convalescence, and emerging zoonotic virus infections may be missed in standard clinic-based testing for febrile illness. In this study, we aimed to serologically assess whether priority paramyxoviruses like NiV and related zoonotic viruses such as MenV had cryptically spilled into human populations in a country with no historical outbreaks.

## 2. Materials and Methods

### 2.1. Sera Collection

Human serum samples were all obtained from previously described cross-sectional prevalence studies conducted among participants presenting with acute undifferentiated febrile illness symptoms at selected health facilities throughout Cambodia between January 2007 and December 2020 [36,37,38]. A total of 1469 convalescent serum samples were selected from 10 provinces, including Battambang (*n* = 3), Kampong Cham (*n* = 132), Kampong Speu (*n* = 318), Kandal (*n* = 211), Kratie (*n* = 489), Phnom Penh (*n* = 126), Preah Vihear (*n* = 5), Ratanakiri (*n* = 58), Stung Treng (*n* = 49), and Svay Rieng (*n* = 108).

### 2.2. Multiplex Microsphere-Based Immunoassay

Human convalescent serum samples (*n* = 1469) were screened for antibodies reactive to the attachment glycoprotein (G) of NiV, HeV, CedV, and GhV, and the hemagglutinin-neuraminidase (HN) surface glycoprotein of MenV in an antigen-based multiplex immunoassay. The G and HN antigens were expressed as native-like tetrameric glycoprotein ectodomains as previously described [39,40]. A mock protein antigen was collected via size exclusion and affinity chromatography from the cell culture supernatant of a stable HEK cell line transfected with an empty expression vector (pcDNA3.1). The mock protein antigen control was incorporated to minimize the influence of antibody reactivity with non-G/HN proteins recovered in the purified supernatant. The mock protein antigen, along with the G and HN antigens, was covalently coupled to magnetic microspheres following the manufacturer’s instructions (MagPlex, Luminex Corporation, Austin, TX, USA). Individual human serum samples were diluted 1:100 in 1× phosphate-buffered saline (PBS) and incubated at room temperature with a master mix of G/HN-coupled beads in 96-well plates with agitation (900 rpm) for 45 min. Subsequently, sample plates were washed (PBS-Tween 20 (0.05%), PBST) three times. Then, a 1:1 mixture of biotinylated-Protein A and biotinylated-Protein G (Thermo Fisher, Waltham, MA, USA) diluted 1:1000 in PBS-T were added to each to detect the presence of antigen-bound IgG. Incubation and washing were repeated as described above before adding streptavidin-phycoerythrin (1:1000 in PBST; Bio-Rad, Hercules, CA, USA) to each well. Plates were analyzed using the xMAP-based MAGPIX system (Luminex Corporation, Austin, TX, USA) and antigen–antibody complexes were measured as the median fluorescence intensity (MFI) of a minimum of 50 unique beads per region. For each microtiter plate, PBS values were subtracted from antigen-specific MFI values.

### 2.3. Cedar Virus Neutralization Test

To investigate whether serum samples containing anti-CedV G IgG binding antibodies contained CedV-neutralizing antibodies, we conducted a virus neutralization test using a replication-competent green fluorescent protein reporter Cedar virus (rCedV-GFP) [41,42]. Human serum samples were serially diluted two-fold from 1:5 to 1:640 in cell growth media (DMEM with 10% cosmic calf serum); then, 50 μL was added to 50 μL of a growth media solution (1:1) containing 80,000 PFU/mL of rCedV-GFP and incubated for two hours at 37 °C. Vero 76 cells were seeded in a black 96-well clear-bottomed plate and allowed to reach 95–100% confluence at 37 °C, 5% CO_2_. For a neutralization positive control, a monoclonal antibody, 14F3, that targets CedV G was similarly serially diluted along with cell culture media that served as the negative control. Cells were washed and then 90 μL of the serum–virus solution was added to each well and incubated at 37 °C, 5% CO_2_ overnight. Cells were then fixed using a 4% formaldehyde solution and scanned, and fluorescent cells were counted using a CTL-ImmunoSpot S6 Universal Analyzer (ImmunoSpot, Cleveland, OH, USA).

### 2.4. Statistical Analysis

All statistics and models were obtained using the statistical software *R* v4.4.3 (R Core Team, 2025) [43]. Key packages were *stats* v3.6.2 (2019) for PCA, *factoextra* v1.0.7 (2020) for PCA and k-medoids clustering [44], *diricletprocess* 0.4.2 (2023) for infinite component Dirichlet process mixture modeling [45], and *mixtools* v2.0 (2022) for finite mixture model analysis [46]. In the absence of control NiV-positive or NIV-negative human sera samples, the R function ‘princomp’ was used to perform a principal components analysis on a data matrix representing the MFI values for each protein antigen. The cumulative proportion of variance for each component was evaluated and it was observed that components 1, 2, and 3 encompassed most of the variation. The silhouette method and elbow method were used to select the k-means number for clustering using the package “factoextra,” with the suggested 2 or 3 clusters. Serological MFI data underwent dimensionality reduction via PCA, followed by k-medoid clustering to identify antigen–antibody sero-reactivity profiles highlighting viral targets for further analysis. These clustering profiles were validated using a Dirichlet process mixture model (DPMM) to confirm that the broad trends were observed in the complex profile of groups generated by an infinite component method, and that rare events were not missed. Following identification of anti-GhV G antibodies by the k-medoid clustering and DPMM, a MFI cutoff value was determined for anti-GhV sero-status using univariate mixture models under the presumption of 2 components representing sero-positive and sero-negative populations with higher and lower mean MFI values, respectively [47,48]. A threshold was established with 90% sensitivity for presumptive sero-positive samples.

Example code is provided via the Open Science Framework and will be found here: https://osf.io/h7ugy/?view_only=93a1f1d5ac244bc0a77c2db4797e4f3e (accessed on 24 June 2025)

## 3. Results

**Figure 1:**
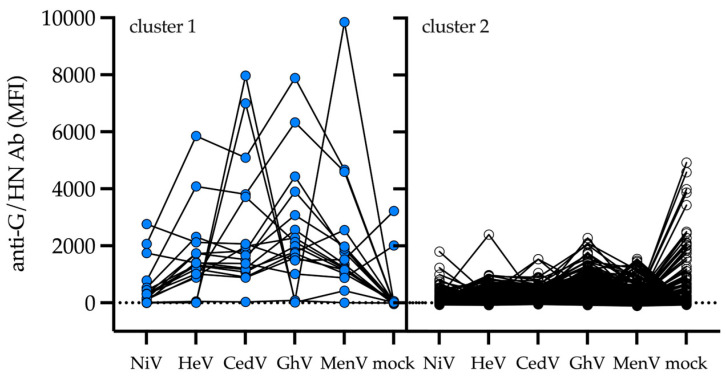
Identification of sero-groups reactive to zoonotic paramyxovirus antigens. Human serum samples (n = 1469) were analyzed by k-means clustering. A two-component model was selected and sub-groups of cluster 1 (*n* = 17)- and cluster 2 (*n* = 1452)-containing samples are shown. Lines connect individual serum samples. MFI, median fluorescence intensity; G, henipavirus attachment glycoprotein; HN, pararubulavirus hemagglutinin-neuraminidase antigen; NiV, Nipah virus; HeV, Hendra virus; CedV, Cedar virus; GhV, Ghana virus; and MenV, Menangle virus.

**Figure 2:**
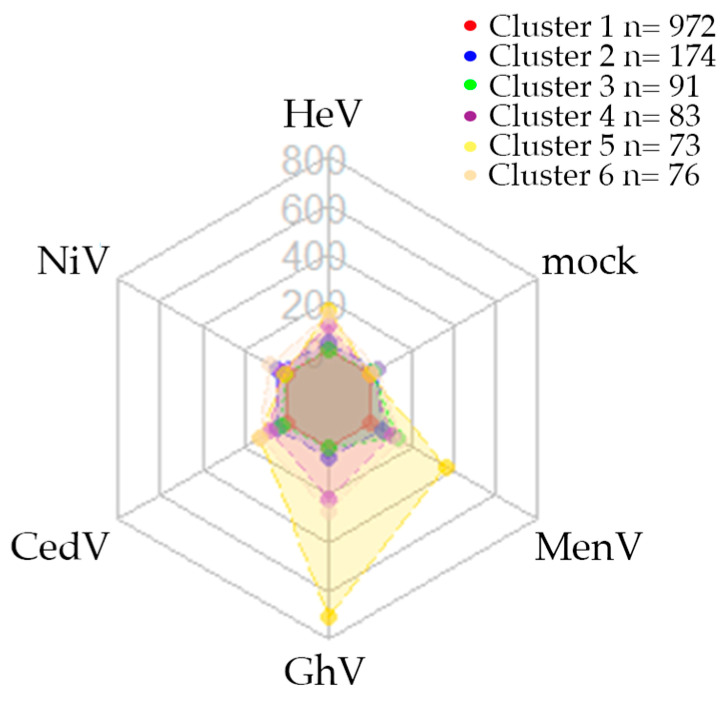
Sero-reactivity profiles of human sera against selected zoonotic paramyxoviruses. Radar charts demonstrating human (n = 1469) sero-reactivity with five paramyxovirus G and HN antigens and a mock control protein. Radial axes represent each of the antigens used as the serological target for detection of antibodies. Scales are a continuous linear measurement of median fluorescence intensity that represent antibody levels. Connecting lines represent the individual clusters based on k-medoids clustering after principal component analysis to six components where cumulative explained variance is >70%.

**Figure A1:**
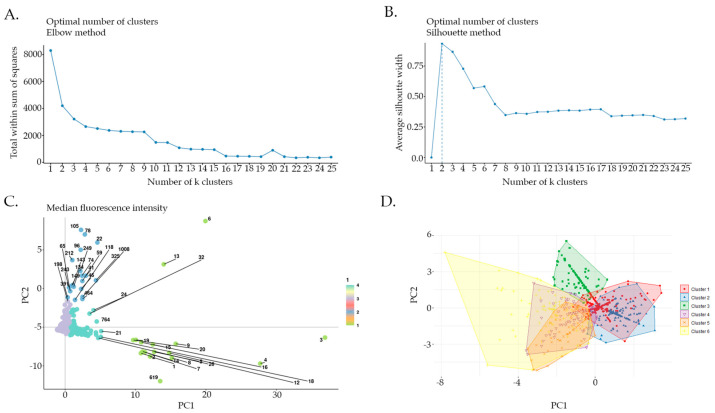
K-means and k-medoids clustering approaches identified distinct groups of sero-reactivity. (**A**) Cluster count optimization by elbow visualization method. (**B**) Validated optimized cluster count by average silhouette width. (**C**) Visualization of data along primary axes of principal components. (**D**) Same data along principal components with cluster boundaries visualized.

We conducted a survey of archived serum samples previously collected under an acute febrile illness study to investigate whether there was evidence of cryptic human exposure to NiV in Cambodia. Antibody responses were first stratified by a two-component model into two clusters likely representing sero-negative (range: 1–1514.25 MFI) and sero-positive (range: 1673.75–9850) data (Figure 1, Figure A1A–D). Assignment to cluster 1 was largely driven by high-magnitude antibody levels against a single virus glycoprotein antigen, e.g., CedV and MenV, or increased magnitudes of co-reactivity with GhV, HeV, and CedV glycoprotein antigens (Figure 1). Assignment to cluster 2 was likely a result of low antibody reactivity with the viral antigens and suggested separation of sero-positive and sero-negative clusters. Next, unique sero-reactivity profiles were identified via k-medoids clustering, permitting the discernment of both discrete and co-reactive groups prior to the determination of sero-positivity. Of note were low-magnitude and diffuse sero-reactivity against GhV (see clusters 4 and 6, Figure 2), and a higher magnitude co-reactive cluster against GhV, CedV, HeV, and MenV (see cluster 5, Figure 2).

**Figure A2:**
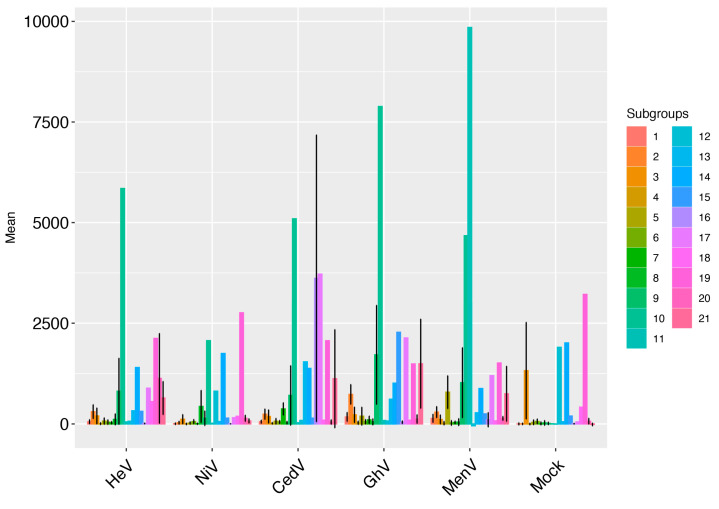
Validation of sero-reactive k-medoid clustering.

**Figure A3:**
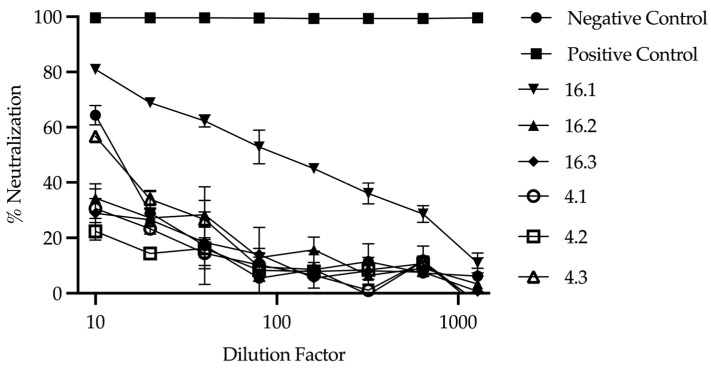
Detection of neutralizing antibodies in Cedar virus-binding antibody positive samples.

These profiles were validated using an infinite mixture model to confirm that these broad strokes were observed in the higher resolution clustering (Figure A2, Table A1). The antisera containing GhV-reactive antibodies displayed varying magnitudes of GhV reactivity (Figure 2), and higher magnitude responses against GhV coincided with co-reactivities to HeV and CedV (see clusters 9, 10, and 15, Figure A2). Antibody binding to an antigen control (mock) was observed as distinct clusters and did not influence the population of putative sero-reactive clusters (see clusters 3, 12, 14, and 19, Figure A2, Table A1). Additionally, we detected antibodies reactive with CedV in six individual serum samples (see clusters 16 and 17, Table 1, Figure A2). Interestingly, one serum sample that we identified as sero-positive for anti-CedV G antibodies possessed CedV-neutralizing antibody titers that were significantly higher than the negative control (IC_50_ = 96.9) (Table 1, Figure A3). Antibody responses to NiV G were seemingly not a driving component of any clusters.

**Figure A4:**
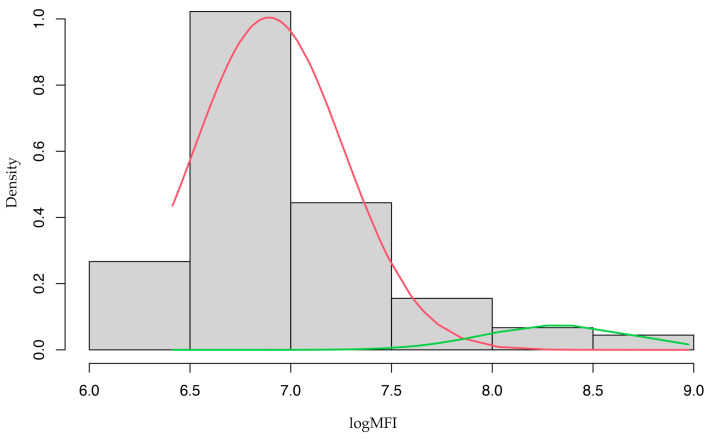
Probability density distributions observed from univariate mixture modeling for threshold analysis of anti-Ghana virus G antibodies. The curves identify the subpopulation with (green) higher MFI values as compared to the subpopulation with (red) lower MFI values. The distinction between these groups is presented as the threshold for seroreactivity.

Following multi-approach detection of anti-GhV antibody levels in this cohort, we next utilized a probabilistic univariate mixture model to determine sero-positivity for GhV. This model presumed sero-negative and presumptive sero-positive sub-populations, and identified six samples of the 32 identified by DPMM within the sero-positive sub-population. A sero-status threshold cutoff of 2582 MFI classified 0.4% (6/1469) of the anti-GhV G distribution as sero-positive (Table A1, Figure A4).

## 4. Discussion

In this study, we investigated whether there was serological evidence of human exposure to NiV and zoonotic paramyxoviruses in Cambodia. We found no serologic evidence of cryptic or subclinical NiV infection in this population of individuals who presented to clinics with acute febrile illness. Instead, we detected antibodies that preferentially bound to the surface attachment glycoprotein of an African henipavirus, GhV, which appears ancestral to the Asiatic henipaviruses, NiV, HeV, and CedV [28], and shares a most common recent ancestor likely able to utilize ephrin-B2 as a virus receptor [49,50]. Furthermore, we surprisingly found serological evidence of a CedV-like virus infection in three participants, including one person with neutralizing antibodies against rCedV. This discovery implied that the infecting virus is closely antigenically-related to CedV, sharing enough epitope similarity in the receptor-binding pocket to generate cross-neutralizing antibodies. In Australia, there has been no documentation of CedV zoonosis. CedV is able to bind to and enter human cells via ephrin-B1 and ephrin-B2 ligands, characteristics of probable zoonotic potential [51]. However, CedV does not express the V and W proteins, key virulence factors for NiV and HeV pathogenesis [52], which provides the molecular mechanism underlying the non-pathogenic nature of CedV infection [21,53,54].

As there are no isolates or replication-competent versions of GhV, we remained limited in our methodological approached to further characterize the binding antibodies detected. Our data suggest that in addition to a diversity of putatively zoonotic shrew-associated henipaviruses in Southeast Asia [55], there is also likely a diversity of henipaviruses ancestral to NiV and HeV, which share epitopes conserved with African henipaviruses, such as GhV [25], including novel henipavirus RNA-dependent RNA polymerase sequences collected from Indian flying foxes (*Pteropus medius*) that form a branch nearest to GhV sequences detected in *Eidolon helvum* bats [56]. Human exposure to African henipaviruses has been identified by sero-surveillance [57], suggesting that these viruses are able to spill into human populations. Lastly, we observed serologic evidence of a MenV-like paramyxovirus infection in a human, and the second Australian bat-borne paramyxovirus after CedV detected in this febrile-illness cohort. Symptomatic human infection by MenV was recorded during the first outbreak in piggeries [33]. MenV has not been detected in Cambodia, and this discovery provides further evidence of an antigenic and likely genetic diversity of zoonotic pararubulaviruses in addition to henipaviruses in Southeast Asia.

Our study has several limitations including a lack of understanding of the exposure history of persons who presented with a febrile illness and zoonotic paramyxovirus IgG sero-status. Additionally, we possessed a limited knowledge of flying fox roosts and proximity to or any contact the study participants may have with flying foxes, or other wildlife. Despite the presence of NiV in a host population (e.g., flying foxes) and human behaviors that could promote transmission, there has never been evidence of an NiV disease outbreak in Cambodia [16,19]. Several flying fox roosts have been recorded in Cambodian provinces, including those from where human serum samples originated. However, we do not retain the level of detail that would permit analysis of proximity to flying fox roosts and integration of spatial dynamics with serological data. Flying foxes are the reservoirs of NiV in Asia, and MenV and CedV in Australia [21,30,58,59], but whether *Pteropus* species in Cambodia are hosts to MenV-like and CedV-like viruses is unknown. Emergence of NiV has been linked to specific anthropogenic behaviors such as the farming of domestic pigs at agriculture sites that overlapped with flying fox roosts in Malaysia [60,61], or the consumption of NiV-contaminated raw date palm sap in Bangladesh [9,62]. Future studies will be critical to identify and characterize animal–human interfaces where spillover may be occurring.

Based on the serologic evidence presented here, we argue for strengthening capacity to detect zoonotic henipaviruses and pararubulaviruses in order to decrease the genomic ambiguity of these serologic findings, and improve our understanding of the pathogenic and pandemic potential of this CedV-like virus in addition to the GhV-like antigenic diversity. Although CedV seems an unlikely zoonotic pathogen, the future spillover of CedV-like henipaviruses that retain the ability to express virulence factors, which suppress host innate antiviral defenses, such as the interferon pathway, may represent novel pathogens and could result in sustained human to human transmission. Without targeted surveillance we will remain unprepared for the emergence of novel paramyxoviruses.

## Figures and Tables

**Table 1:** Antigen–antibody median fluorescence values for serum samples identified in anti-Cedar virus sero-reactive clusters.

Cluster.ID	GhV	HeV	CedV	NiV	MenV	mock	VNT ^2^
**16.1** ^1^	11	7	7968	−0.5	422.25	9.5	+
**16.2**	77.5	35	6997.5	−2	5	−9.5	−
**16.3**	85	−0.25	1533	−1	6	−5.5	−
16.4	16.5	14.75	1051.25	13	15.5	10.25	
16.5	18.75	3.75	532.5	7.5	67.25	28	
17.1	2134.25	886.25	3720.75	155.25	1197.75	51	
***4.1*** ^3^	12.5	2	13.25	24.75	9	1.75	−
***4.2***	−0.5	−7	0.5	−4.5	−23.25	−21	−
***4.3***	10	6.75	10	7	37	9.75	−

^1^ Bold type face indicates samples that were tested for anti-Cedar virus neutralization activity. ^2^ VNT, virus neutralization test; + positive; and − negative. ^3^ Italicized type face indicates samples that were not identified as anti-CedV G sero-reactive and used as negative sample controls in the VNT. Cluster.ID references individual serum samples.

## Data Availability

The datasets presented in this article are not readily available because they are the property of the United States Navy. Reasonable requests to access the datasets will be considered on a case-by-case basis and should be directed to the corresponding author.

## Disclaimer

Jeffrey Hertz (LCDR, USN, MSC) and Andrew G. Letizia (CAPT, USN, MC) are military Service members. Eric D. Laing is a federal employee within the Department of Defense. This work was prepared as part of their official duties. Title 17, U.S.C., §105 provides that copyright protection under this title is not available for any work of the U.S. Government. Title 17, U.S.C., §101 defines a U.S. Government work as a work prepared by a military service member or employee of the U.S. Government as part of that person’s official duties. The views expressed in the article are those of the authors and do not necessarily express the official policy and position of the Uniformed Services University of Health Sciences, Henry M. Jackson Foundation for the Advancement of Military Medicine, U.S. Navy, the Department of Defense, the U.S. government, or any of the other institutions affiliated with any of the authors.

## Abbreviations

The following abbreviations are used in this manuscript:

NiV	Nipah virus
HeV	Hendra virus
CedV	Cedar virus
Ghana virus	GhV
Menangle virus	MenV
MMIA	Multiplex microsphere immunoassay
G	Henipavirus attachment glycoprotein
HN	Pararubulavirus hemagglutinin-neuraminidase protein
xMAP	Multi-analyte profiling
PBS	Phosphate-buffered saline
PBST	Phosphate-buffered saline, Tween-20 (0.05%)
rCedV	Recombinant Cedar virus
DPMM	Dirichlet process mixture model
PCA	Principal component analysis

## Appendix A

**Table A1:** Antigen–antibody median fluorescence intensity values for each virus glycoprotein identified in anti-GhV G and anti-MenV HN sero-reactive clusters.

Cluster.ID	GhV	HeV	CedV	NiV	MenV	Mock
**9.1**	6331	4084.25	3808.75	779	4600	−22.5
**9.2**	4431	1730	1820.5	145.5	1776	−3.75
**9.3**	3077.5	2317.75	1662.25	526.5	1975.5	46.25
**9.4**	3903.5	1740	1519.75	305.5	1900	−38
**9.5**	2559.5	1412	1208.5	54.5	1349.5	−25.25
9.6	1785.75	1322.75	1166.5	448	2553.75	20.25
9.7	1987.25	1307.5	1079.5	309.25	1140.5	10.5
9.8	2041.5	930.75	901	57.5	782	13.5
9.9	1735.25	1187.5	901	314.75	1025	46.75
9.10	1577	1014.5	886.75	306.25	1154	56.5
9.11	1564.5	975	822	203.5	980.25	81.25
9.12	1700.75	959.75	648.5	58	690.75	23.75
9.13	2089.75	657.25	598.5	62	1154	13.5
9.14	1438.75	400.75	582.5	26	951	34.75
9.15	1311.25	652.75	567	183.5	697.5	11.5
9.16	1474.75	719.5	531	146.25	1205.25	100.75
9.17	961.25	659.875	516.875	187.5	529.625	64.75
9.18	658.75	336.25	480.5	43.5	586.25	−14
9.19	1742	354.75	325	17	597.5	28.75
9.20	1130.75	359	323.75	67	683.75	−32.75
9.21	1199.5	354.25	293.5	85.5	1342.75	14
9.22	934.75	330.25	289.5	36.25	702	8
9.23	1068.25	256.75	246	59.5	982	−33.25
9.24	1772.25	481.25	240.25	−11.5	143	−19.5
9.25	808.5	423.25	229	−21.5	-29	−26.25
9.26	999.25	115.75	228.5	7.5	379	14
9.27	999.25	115.75	228.5	7.5	379	14
9.28	999.25	115.75	228.5	7.5	379	14
9.29	663.25	115.5	100.5	15.5	7	−41.5
9.30	559.5	64	72	7	984	74.75
9.31	757.75	−14.25	−2.5	−18.5	255.5	7
**10.1**	7883.25	5847.75	5095	2065.5	4673	4
11.1	84	50	30	17	9850	−4
15.1	2275	311.75	144.25	144.75	249	196

Underlined, bold type face indicates samples that were deemed sero-positive by a probabilistic latent class analysis. Deidentified specimen labels were converted to sequential numbers after cluster assignment. Cluster.ID references individual serum samples.

Principal component analysis (PCA) visualization of clusters along primary principal components. Sero-reactivity profiles can be identified as groups defined by distinctly separate centers. (A) Elbow method to identify the optimal number of k-means clusters. (B) Silhouette method to identify the optimal number of k-mean clusters. (C) Principal component analysis of the two cluster, k-means analysis shows poor separation with high-volume clusters spread to include outlier range data. (D) Six-cluster k-medoids analysis shows greater separation even without accounting for higher dimensionality with less outlier influence on cluster centers. The centers of these clusters are used to define each cluster’s representative seroreactivity as seen in Figure 1, using the same cluster-color assignments.

Dirichlet process mixture modeling was used as an infinite mixture modeling technique to identify greater discrete separation between potential sero-reactivity profiles. The higher response to Cedar virus (CedV), Ghana virus (GhV), and Menangle virus (MenV) can be seen separately from the diffuse low-level reactivity to Hendra virus (HeV) and Nipah virus (NiV). CedV-specific reactive clusters can be identified as 16 and 17, while GhV clusters are 9, 10, and 15. A single highly reactive serum sample against MenV HN can be found in cluster 11.

Three anti-Cedar virus positive samples (closed symbols) and three anti-Cedar virus negative samples (open symbols) were tested for the presence of neutralizing antibodies against a replication-competent recombinant Cedar virus. The anti-CedV G monoclonal antibody 14F3 positive is indicated by a closed square and the cell culture media negative control is indicated by a closed circle.

Log transformed median fluorescence intensity (MFI) values for Ghana virus G were visualized as a product of two presumptive groups (sero-positive and sero-negative) after zero inflation using the distribution of mock MFI values to probabilistically remove noise. A 90% sensitivity cutoff was calculated for the group with the higher mean (presumptive sero-positive).
